# ΔNp63 Is Essential for Epidermal Commitment of Embryonic Stem Cells

**DOI:** 10.1371/journal.pone.0003441

**Published:** 2008-10-17

**Authors:** Alain Medawar, Thierry Virolle, Philippe Rostagno, Stéphanie de la Forest-Divonne, Karen Gambaro, Matthieu Rouleau, Daniel Aberdam

**Affiliations:** 1 INSERM, U898, Nice, France; 2 Université de Nice-Sophia Antipolis, Nice, France; 3 INSERTECH, Technion, Haifa, Israel; Baylor College of Medicine, United States of America

## Abstract

*In vivo* studies have demonstrated that p63 plays complex and pivotal roles in pluristratified squamous epithelial development, but its precise function and the nature of the isoform involved remain controversial. Here, we investigate the role of p63 in epithelial differentiation, using an *in vitro* ES cell model that mimics the early embryonic steps of epidermal development. We show that the ΔNp63 isoform is activated soon after treatment with BMP-4, a morphogen required to commit differentiating ES cells from a neuroectodermal to an ectodermal cell fate. ΔNp63 gene expression remains high during epithelial development. P63 loss of function drastically prevents ectodermal cells to commit to the K5/K14-positive stratified epithelial pathway while gain of function experiments show that ΔNp63 allows this commitment. Interestingly, other epithelial cell fates are not affected, allowing the production of K5/K18-positive epithelial cells. Therefore, our results demonstrate that ΔNp63 may be dispensable for some epithelial differentiation, but is necessary for the commitment of ES cells into K5/K14-positive squamous stratified epithelial cells.

## Introduction

During embryonic development, the body surface is first covered by a single layer of basal epithelial cells. As development proceeds, this primitive epithelium switches on a differentiation program leading to stratification: the ectodermal cytokeratins K8/K18-positive cells of the primitive epithelium are replaced by basal epidermal K5/K14-positive cells, and progressively, several layers of suprabasal differentiated cells appear offering an efficient resistance against environmental stress. In the adult skin epidermis, the stratified squamous epithelium maintains a basal layer of proliferating cells that gives rise to multiple layers of terminally differentiating cells that continually reach the body surface from where they are shed. Epidermal stem cells located interfollicularly and within the bulge region of the hair follicles retain a self-renewing capacity, critical to preserve the tissue integrity during the life of the organism. The molecular mechanisms inducing the stratification program and those regulating the self-renewing process still remain to be clearly defined.

The p63 transcription factor, a homologue of the tumour suppressor gene p53 [1], is characterized by six different isoforms under the control of two distinct promoters and three different alternative splicings leading to the production of ΔN- or TA-p63, and alpha, beta or gamma isoforms, respectively. Recently p63 has been implicated in both the development and the maintenance of stratified epithelia [2]–[4]. In loss-of-function models generated by two different groups, mice lacking p63 have been shown to be severely compromised in their ability to generate an epidermis, many other stratified epithelia as well as the limbs [2], [3]. Recent reports convincingly demonstrated the implication of p63 in epithelial stem cell renewal potential, as the absence of p63 was shown to induce a loss of proliferative potential of epidermal [5], [6], corneal [7] or thymic epithelial cells [6], [8]. On the other hand, experiments to complement the absence of p63 during development were designed by crossing p63-null mice with transgenic mice expressing different isoforms of p63 under the control of a K5-promoter [9]. Those mice expressing TAp63 and ΔNp63 developed patches of epidermal differentiated cells, but the overall rescue of the epidermal phenotype remained partial, suggesting an earlier role of p63 during epidermal morphogenesis, before the K5-promoter is activated. Accordingly, Koster et. al showed that the exogenous expression of p63 in single-layered lung K8/K18-positive epithelial cells was able to initiate a stratification program, with formation of K14-positive cells [10], suggesting that p63 is required for the initiation of epidermal stratification, a process mediated in part by the direct induction of AP-2γ expression [11]. However, the precise role of p63 in the morphogenesis of epidermal cells remains to be defined.

Embryonic stem (ES) cells, derived from blastocyst-stage early mammalian embryos, are pluripotent and can initiate lineage-specific differentiation programs of many tissues and cell types *in vitro*. ES cells are thus an effective tool in recapitulating *in vitro* the main steps of embryonic development. We have recently reported the efficient derivation of ectodermal and K5/K14-positive epidermal cells from murine ES cells [12] and clarified the molecular mechanism of BMP-4 in the neuroectodermal fate [13]. In the present study, we show that the ΔNp63 isoform is expressed soon after BMP-4 treatment, along with the simultaneous expression of direct or indirect p63-target genes associated with epidermal morphogenesis. Furthermore, p63 knockdown drastically inhibits the progression of ES cells along the pluristratified epithelial fate, while de novo expression of p63 is compulsory for pluristratified epithelial commitment once the ectodermal stage has been reached. Finally, ectopic expression of ΔNp63 in ES-derived ectodermal cells is able to induce their commiment. Therefore, our results demonstrate that ΔNp63 is absolutely necessary at the ectodermal stage for the proper commitment of ES cells into K5/K14-positive epithelial cells, and therefore suggest that ΔNp63 function is required for the embryonic development of pluristratified squamous epithelia.

## Results

### ΔNp63 is expressed during epithelial differentiation of mouse ES cells

We have previously established a cellular model that recapitulates *in vitro*, the main steps of embryonic neuroectodermal fate (Fig. S1). ES cells seeded on a layer of fixed-NIH-3T3 cells in a serum free medium differentiate into neuroectodermal precursors, while early mesodermal and endodermal commitments are inhibited [13], [14]. These neuroectodermal precursors have been shown to further differentiate in 7 to 10 days into neural cell populations [14], [15]. When treated with BMP-4 from day 3 to 5, the neural differentiation process is efficiently inhibited, partially through the apoptosis of Sox-1^+^ neural precursor cells, as we recently demonstrated [13], allowing the remaining committed cells to differentiate into K18-positive ectodermal cells, which are the putative precursors of epithelial cells. Addition of serum at day 9 allowed keratinocyte differentiation to proceed further [13]. Epidermal differentiation proceeds through successive steps that lead proliferative keratinocytes, positive for cytokeratins K5 and K14, to undergo terminal differentiation with the appearance of specific markers like K1, K10 and involucrin.

**Figure 1:**
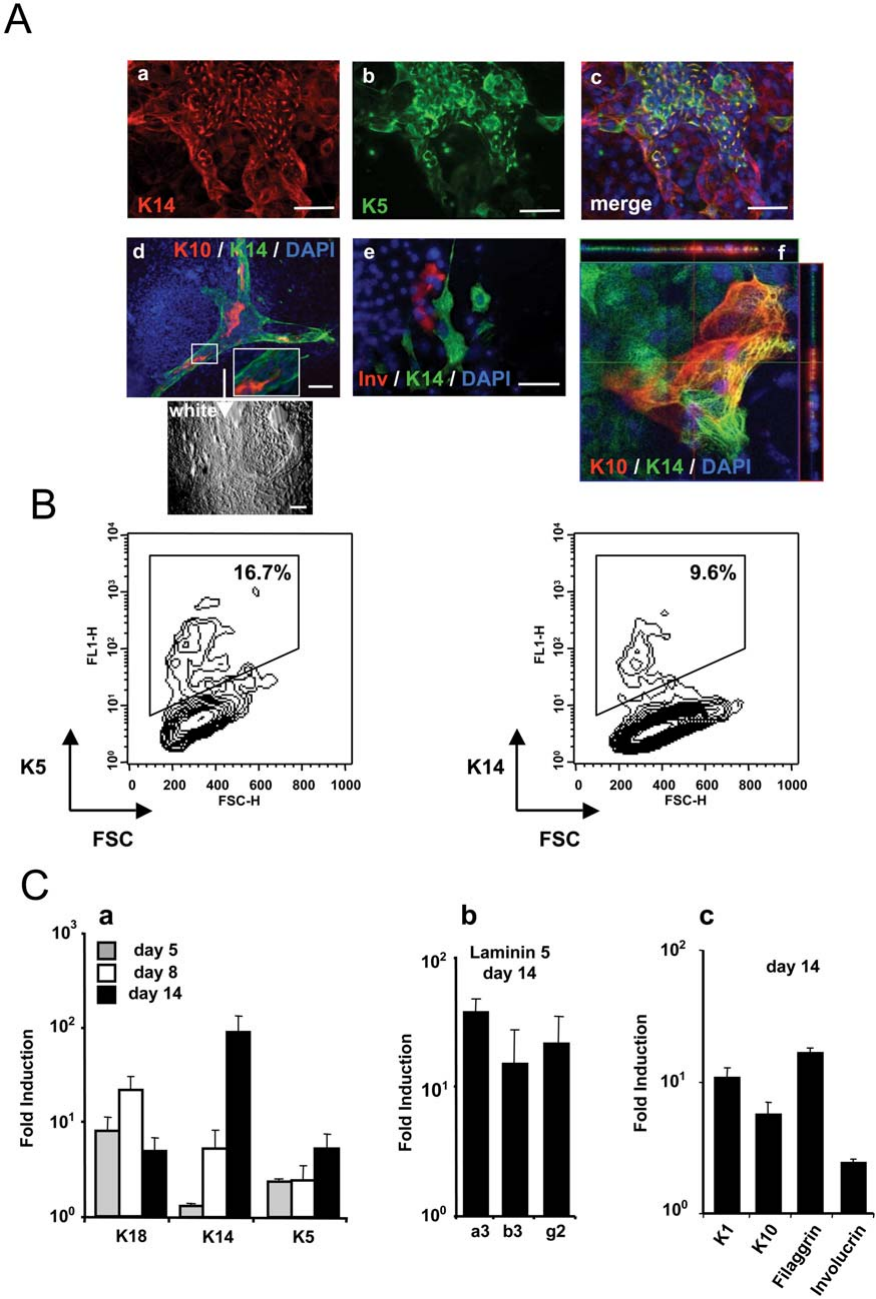
Epidermal differentiation of murine ES cells. (A) Appearance of K5^+^/K14^+^ and involucrin-positive epidermal cells from mouse ES cells treated with BMP-4 and serum. The differentiated cells were immunostained for cytokeratin K14, K5, K10 or involucrin at day 14 (panels a–e). Scale bars = 50 µm. Confocal analysis of differentiated cells (at day 14) (panel f). (B) Flow cytofluorimetry analysis of K5 and K14 on differentiated cells at day 14 of culture. The graphs represent the fluorescence intensity for the indicated proteins plotted against the cell size (forward scatter, FSC). The percentages of positive cells among the gated populations are indicated on graphs. (C) Gene expression was analyzed by real-time RT-PCR, for K18, K5, and K14 at the indicated differentiation time points (panel a), for laminin-5 chains at day 14 (panel b), and for K1, K10, filaggrin and involucrin at day 14 (panel c). The value for each gene was normalized to untreated control ES cultures, and represents the average of three independent experiments±sd.

During ES cell differentiation, K5/K14-positive epithelial cells were detected by day 10 /11 and further proliferated into large patches by day 14 (Fig. 1-A panels a–c). FACS analysis indicated that, among the differentiated cells, about 10% became epidermal cells at day 14 (Fig. 1-B). The terminal differentiation events occured as K10-positive cells as well as involucrin-positive cells were also detected by immunofluorescence analysis (Fig. 1-A, panels d,e). These cells were in close contact to patches of K5/K14-positive cells (Fig. 1-A, panels d,e) suggesting that the stratification program was efficiently initiated during ES differentiation. Confocal analysis clearly confirmed that the differentiated K10-positive cells were different from the proliferating K14-positive cells (Fig. 1-A, panel f). Real-time Q-PCR (qRT-PCR) analysis was performed at different time points: day 5 (48 h after BMP-4 treatment), day 8 (24 h before serum addition) and day 14 (end of the culture) (Fig. 1-C). Transcription of the K18, K14 and K5 genes was induced after BMP-4 treatment (days 5 and 8). Following serum addition (day 14), K18 gene expression was reduced while transcription of K5 and K14 genes were enhanced (Fig. 1-C, panel a). A detailed kinetic experiment for K18 and K14 gene expression confirmed the progression along the epithelial developmental process (Fig. S2-A). The transcription of the three genes encoding laminin-5, the major adhesion ligand of keratinocytes, was also clearly induced during this epithelial differentiation process (Fig. 1-C, panel b). At day 14, specific markers of terminal differentiation of stratified epithelia (K1, K10, involucrin and filaggrin) were also detected (Fig. 1-C, panel c). These results indicate that this ES cell model recapitulates *in vitro* the stepwise appearance of stratified epidermal cells and is therefore suitable to study the function of p63 isoforms during embryonic epithelial cell differentiation.

**Figure 2:**
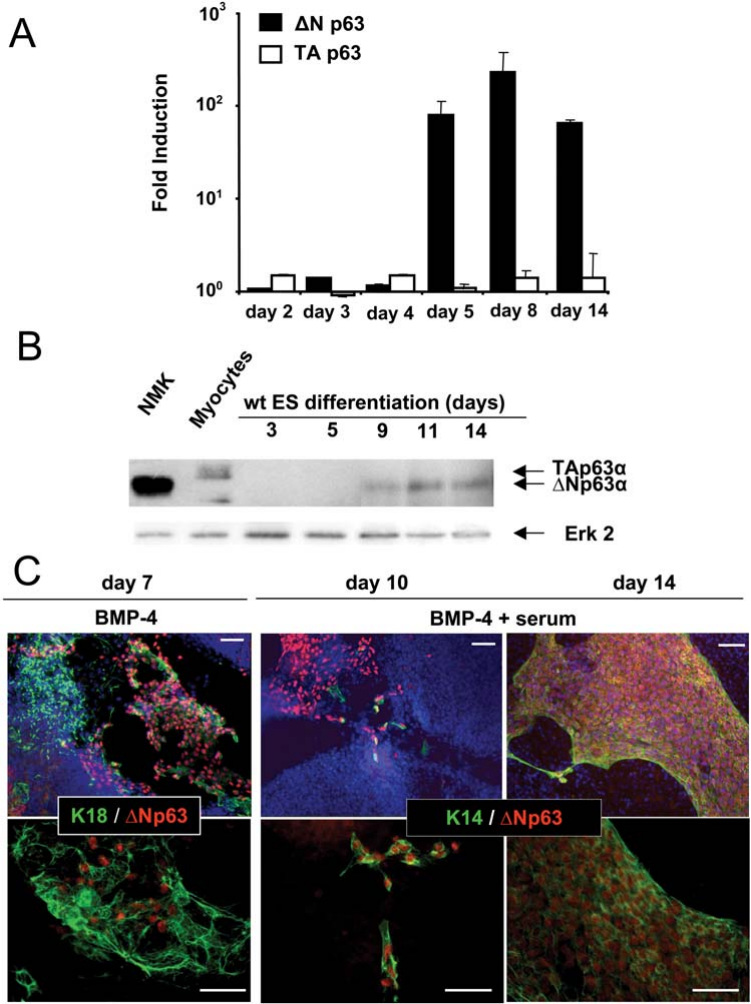
Expression of ΔNp63 during epidermal differentiation of murine ES cells. (A) Real-time RT-PCR analysis for ΔN- and TA-p63 isoform gene expression at the indicated differentiation time points. The value for each gene was normalized to untreated control ES cultures, and represents the average of three independent experiments±sd. (B) Immunoblots of endogenous p63 isoforms in normal mouse keratinocytes (NMK), myocytes and ES cells during 3, 5, 9, 11 and 14 days of differentiation, as revealed by anti-p63 and anti-Erk2 (as loading control) antibodies. Normal mouse keratinocytes (known to express endogenous ΔNp63 isoforms) and differentiating myocytes (which have been shown to express the TAp63 isoform, [24]) served as molecular weight references. (C) Immunofluorescence detection of ΔNp63 during ES cell differentiation. The differentiating cells were immunostained for K18 or K14 (green) and for ΔNp63 (red). Dapi staining is shown in blue. Scale bars = 50 µm.

ΔNp63 transcripts were detected after BMP-4 addition at day 5 and remained high during ES differentiation (Fig. 2-A and Fig. S2-B). TAp63 was never detected during epidermal commitment. By Western blot, ΔNp63 was detectable at days 9, 11 and 14 (Fig. 2-B and Fig. S2-C). In accordance with the qRT-PCR analysis, TAp63 protein was never detected during ES differentiation. Several non specific bands, which are characteristic of the pan-p63 antibody used in this study, were observed but their migration rates did not correspond to any p63 isoform (Fig. 2-B and Fig. S2-C).

Of note, ΔNp63 isoform was detected by Western blot analysis much later (day 9) than its transcript (day 5), probably due to the heterogeneity of the cell population and the difference of sensitivity of the two methods. Therefore, we evaluated p63 detection at the cellular level by immunofluorescence staining. In the absence of BMP-4, no ΔNp63 expression could be observed at any time point. In the presence of BMP-4, nuclear-stained ΔNp63-positive cells became detectable among K18-positive ectodermal cells by immunofluorescence staining at day 7 (Fig. 2-C). Further addition of serum allowed ΔNp63-positive cells to differentiate into K14^+^/K5^+^ keratinocytes, all K14-positive cells being then ΔNp63-positive (Fig. 2-C). At day 14, large patches of K14/K5/ΔNp63-positive cells were observed. It should be noted that not all ΔNp63-positive cells were positively stained for K14, while all K14^+^ cells expressed ΔNp63 (Fig. 2-C) It suggests that these ΔNp63-positive cells were not yet committed into the keratinocyte lineage, or that part of them were already committed to alternative epithelial fates. Finally, we were not able to detect TAp63-positive cells using a specific antibody, confirming the absence of TAp63 transcripts (data not shown).

### ΔNp63 expression is required for the commitment of mouse ES cells into keratinocytes

**Figure 3:**
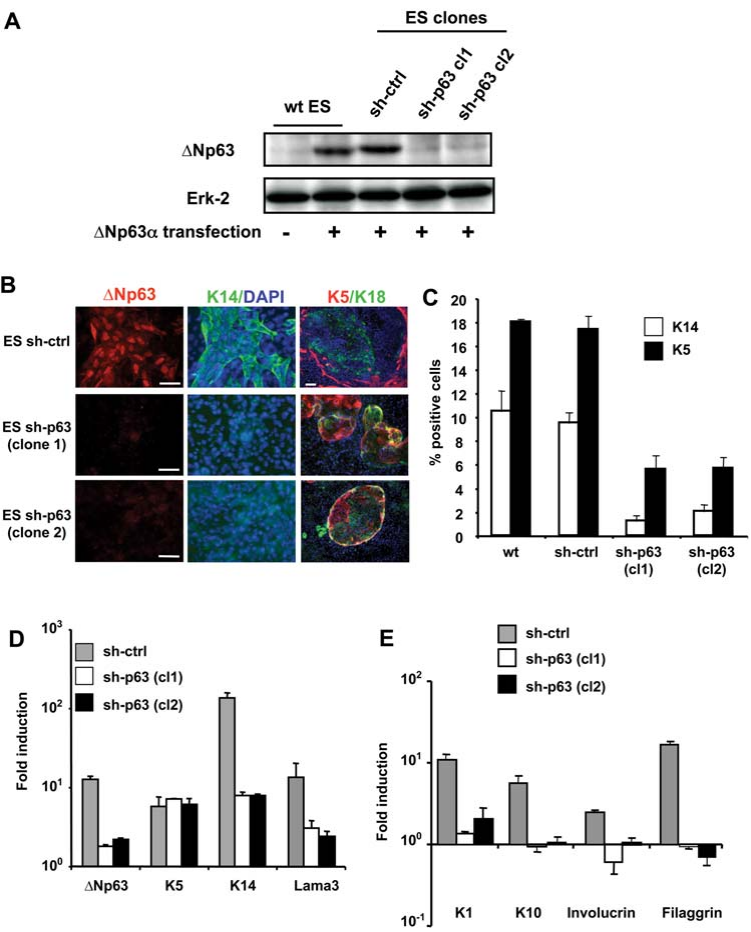
p63 is required for epithelial differentiation of ES cells. Stable ES cell clones in which ΔNp63 gene expression has been inhibited with shRNA, were produced and induced to differentiate into the epidermal fate. (A) Analysis of ES clones transfected with a control pSuper sh-RNA (ctrl) or a pSuper sh-RNA specific for p63 (clones sh-1, sh-2). Wild type ES cells and stable clones were transiently transfected with a ΔNp63-expressing vector and analyzed 48 h later by Western blot with anti-ΔNp63 and anti-Erk2 (as loading control) antibodies. The two p63 sh-RNA clones displayed a strong inhibition of the ectopic ΔNp63 expression while the pSuper control was unable to regulate it. (B) Immunofluorescence staining for the indicated proteins in control and sh-p63 ES cell clones at day 14 of differentiation. Dapi staining is shown in blue. Bars = 50 µm. (C) Percentages of K14- or K5-positive cells differentiating from wt-ES cells, control-sh or p63-specific sh ES clones at day 14 determined by flow cytofluorometric analysis. (D) Real-time RT-PCR analysis at day 14 of differentiation for ΔNp63, K5, K14 and Lama3 (left panel) and for K1, K10, filaggrin and involucrin (right panel) genes. ES cell clones expressing either a control- or p63-specific shRNA were tested.

To evaluate whether ΔNp63 was required for stratified epithelial commitment, stable ES cell clones that express a small hairpin (sh) RNA inhibiting p63 expression were produced. As shown in Fig. 3A, two independent ES cell clones (sh-p63-cl1 and sh-p63-cl2) displayed, by Western-blot analysis, a strong inhibition of exogenously expressed ΔNp63, which was not observed with the sh-control clone. Upon differentiation with BMP-4 and serum, the sh-p63 clones produced, at day 14, a number of ectodermal K18-positive cells similar to the wild type control cells (Fig. S3-A, B). It confirmed that the ectodermal differentiation of ES cells was independent of the expression of ΔNp63. However, while large patches of K14-positive cells were observed within the sh-control clone (Fig. 3-B), very few isolated K14-positive cells were detected with sh-p63-cl1 and sh-p63-cl2 clones at day 14. Analysis by FACS confirmed that the absence of ΔNp63 resulted in a strong reduction of K14 and K5-expressing cells at day 14 (Fig. 3-C). At the transcriptional level, we further confirmed the strong inhibition of the K14 mRNA level in differentiating sh-p63-ES clones (Fig. 3-D). Accordingly, lama3 gene expression, as well as terminal differentiation markers such as K1, K10, involucrin and filaggrin were completely repressed when p63 was knocked down (Fig. 3E). The two other genes encoding for laminin-5 (lamb3 and lamc2) were repressed as well in sh-p63 ES cells (data not shown). Interestingly, while K14 expression was drastically reduced, the expression of K5 mRNA was not modulated by the absence of ΔNp63 and a significant percentage of K5-positive cells was still observed by FACS analysis (Fig. 3-C) with the two sh-p63-ES clones (5.6% and 5.7%, respectively), as compared to sh-control and wt-ES cells (17.4% and 18.0%, respectively). In the absence of K14, K5-positive cells were not able to produce keratinocytes and accordingly we could no longer observe the patches of K14/K5-positive cells obtained during the differentiation of control ES cells (Fig. 3-B). Instead, the K5-positive cells, orphans of K14, were systematically detected within structures of rounded flat clones still positive for the K18 ectodermal marker (Fig. 3-B). Actually, this particular structure was also detectable within wt and sh-control ES cell differentiation along with the large K14/K5-positive patches. Since they were detected as early as day 11 and still present at the end of the culture (up to day 17), they may represent alternative epithelial cells.

### Activation of p63-regulated or-dependent genes during epidermal differentiation

**Figure 4:**
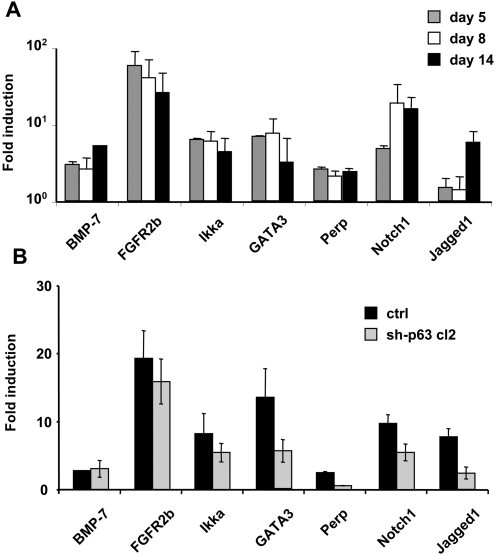
Time course of relative mRNA expression of several p63-regulated genes. (A) Real-time RT-PCR analysis for p63-targets genes at 3 differentiation time points, normalized to untreated control ES cultures. The data represents the average of three independent experiments±sd. (B) Relative mRNA expression for different p63-regulated genes. (A) Real-time RT-PCR analysis for p63-target genes at 3 differentiation time points, normalized to undifferentiated ES cells at day 0. (B) Real-time RT-PCR analysis for p63-target genes in control and sh-p63 cl2 ES cells at day 14 normalized to undifferentiated WT ES cells at day. The data represents the average of three experiments±sd.

Recently, several genes dependent on p63 expression and associated with the murine early embryonic epidermal morphogenesis have been identified [16]–[18]. Among them, BMP-7, FGFR2b, Notch1 and Jagged1 transcripts have been shown to be absent from the developing epidermis of p63-deficient mice [18]. IKKα and GATA3 are two other genes regulated by p63 and associated with epidermal development [16], [19], [20]. Finally, Perp, a protein promoting the stable assembly of desmosomal adhesive complexes, was one of the first described p63 regulated proteins implicated in epidermal development [17]. We thus monitored their expression during ES epidermal differentiation as compared to undifferentiated ES cells. Upon BMP-4 treatment, in parallel to the induction of ΔNp63 gene expression, all the transcripts were detected by qRT-PCR (Fig. 4A). The relatively low activation of some of them (2–5 fold) as compared to the very pronounced induction of ΔNp63 (100 fold) reflected that ΔNp63 was not expressed at all before BMP-4 treatment (>35 cycles of qPCR) while mRNA of these genes were already detected before (22–25 cycles). To demonstrate that p63 is directly responsible for at least part of this modulation, the expression of potential p63-target genes were tested at day 14 in sh-63-cl2 ES cells as compared to control ES cells. Absence of p63 drastically reduced the expression of each gene during epidermal commitment, with the exception of BMP-7, which could be activated by an alternative mechanism, independent of the epidermal commitment (Fig. 4B). Similar results were obtained with the second sh-p63-cl1 (data not shown). We can conclude that the present model of ES-derived stratified epithelium commitment faithfully recapitulates molecular events known to be essential in epidermal morphogenesis.

### Exogenous expression of ΔNp63 does not allow the production of K14-positive cells in absence of BMP-4 and serum

**Figure 5:**
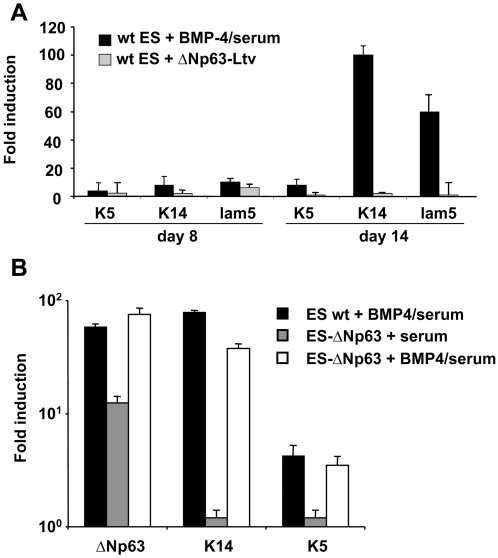
Ectopic expression of ΔNp63 does not induce efficient stratified epithelial commitment of ES cells. (A) Differentiating ES cells were either treated with BMP-4 (at day 3) or transduced (at day 4) with lentiviruses expressing either ΔNp63 or irrelevant proteins (red tomato or Pax6) as negative controls. Real-time RT-PCR analysis for K5, K14 and laminin-5 gene expression was performed at days 5, 8 and 14 of ES cell differentiation. The value for each gene was normalized to either untreated control cultures for BMP-4-treated ES cells or red tomato transduced control cultures for p63-transduced ES cells. The values represent an average of three determinations±sd. (B) Wild type ES or ES cells stably expressing ΔNp63 were induced to differentiate with and without BMP-4. Real-time RT-PCR analysis for K5, K14 and p63 gene expression was performed at day 14. The value for each gene was normalized to untreated wild type ES cells at day 14. The values represent an average of three determinations±sd.

Since ΔNp63 expression was detected at day 7, before the appearance of K14-positive cells, we evaluated whether exogenous expression of ΔNp63 could, in the absence of a BMP-4 dependent signalling pathway, induce keratinocyte commitment. ES cells were transduced, at day 4 of differentiation, with a lentiviral vector expressing ΔNp63. At days 8 and 14, expression of keratinocyte-specific gene was barely detected after infection with ΔNp63-lentivirus, as compared to cells cultivated in the presence of BMP-4 and serum (Fig. 5A). Careful systematic counting of large areas under the microscope detected very few K14^+^ cells (data not shown). Thus, ΔNp63 is not able to induce keratinocyte differentiation in absence of BMP-4. To define if ΔNp63 can synergize the effect of BMP-4, a ΔNp63-expressing construct was stably inserted into ES cells which were induced to differentiate with and without BMP-4. As illustrated in Fig. 5B, no enhancement of K14^+^ or K5^+^ cell production was detected by qRT-PCR when the ES cells were treated with both BMP-4 and ΔNp63 as compared to BMP-4 alone. Furthermore, infection of ES cells with a lentivirus expressing ΔNp63 at day 4 of differentiation did not enhance the effect of BMP-4 on keratinocyte production (data not shown). Since we previously have shown that BMP-4 efficiently induces ES cell to differentiate into ectodermal K8^+^/K18^+^ cells ([13] and Fig. S2-A), it suggested that ΔNp63 can induce epidermal commitment only in ES-derived cells that already have received and integrated ectodermal signals delivered by BMP-4.

### ΔNp63 expression promotes committed ectodermal cells to differentiate into keratinocytes

**Figure 6:**
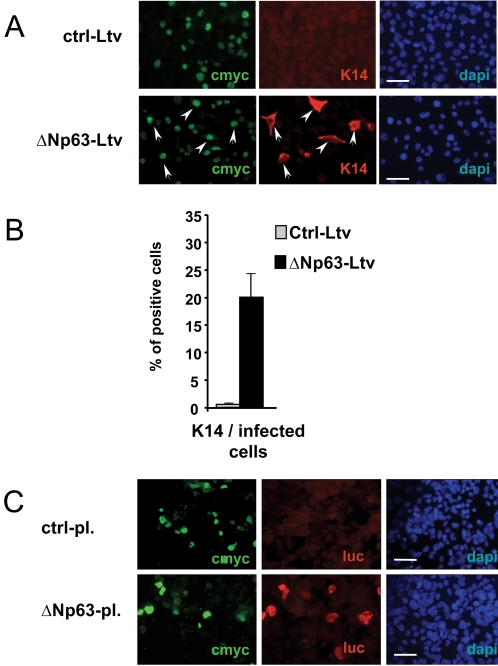
ES-derived ectodermal cells differentiate into K14/K5-positive epidermal cells upon ΔNp63 ectopic expression. An ectodermal K8^+^/K18^+^ cell line was derived by serial clonal dilutions of differentiating ES cells after BMP-4 treatment. (A) Immunofluorescence staining of the ES-derived ectodermal cell line 48 h after infection with a lentivirus expressing c-myc-tagged-ΔNp63. Control cells were transduced with a control c-myc-tagged-pax6 lentivirus. Nuclei are stained with DAPI. Bars = 50 µm. (B) Percentages of K14-positive cells amongst transduced cells 48 h after infection, as determined by immunofluorescence staining of c-myc and K14-positive cells. (C) Immunofluorescence staining of the ES-derived ectodermal cell line 48 h after cotransfection of c-myc-tagged-ΔNp63 and a p63-responsive reporter luciferase construct. Positive cells were detected with c-myc and luciferase antibodies. Nuclei are stained with DAPI. Bars = 50 µm.

To further demonstrate that ΔNp63 is potent to induce ectodermal precursors to become keratinocytes, we isolated by serial dilution of BMP-4-treated ES cells at day 7, a homogenous ectodermal cell line positive for K8/K18 cytokeratins [25]. Exogenous expression of ΔNp63 in this ectodermal cell line indeed induced the differentiation of ΔNp63-transduced cells into K14-positive cells (Fig. 6-A and B). None of the control-transduced cells expressed the keratinocyte-specific genes (Fig. 6-B). Since not all (about 25–40%) of the ectodermal cells expressing *de novo* ΔNp63 became K14^+^-cells, we hypothesized that the ES-derived ectodermal K18^+^ cell population could be heterogeneous in its response to p63. To test if p63 still remains able to transactivate in these unresponsive cells, the ES-derived ectodermal cell line was cotransfected with ΔNp63 and a p63-responsive reporter construct [26]. 48 h after transfection, cells were analyzed by immunofluorescence staining for tagged-p63, luciferase and K14 gene expression (Fig. 6C). Interestingly, although not all the p63-transfected cells became K14^+^ keratinocytes, they all expressed luciferase, strongly suggesting that the K18^+^ ectodermal cells unable to become K14^+^ cells are locked by an unknown mechanism. Further studies will be necessary to identify the molecular differences between cells belonging to this apparent homogenous ectodermal cell population. Finally, p63-target genes were also induced upon ectopic expression of ΔNp63 (not shown, Rostagno et al, in preparation). Altogether, these results indicate that ΔNp63 is necessary to switch-on the differentiation program of pre-committed ectodermal cells into K14-positive epithelial cells.

## Discussion

The ability of ES cells to remain pluripotent and to differentiate *in vitro* in directed or selected cell fates, depending on culture conditions, make them a powerful tool to investigate *in vitro* molecular events involved in embryonic development. Here, we demonstrated that ΔNp63 is a key factor necessary for embryonic epidermal differentiation. In absence of p63, committed ectodermal cells are not able to produce neither proliferating (K14^+^/K5^+^) nor terminally differentiated (K1^+^/K10^+^) keratinocytes. The implication of p63 in pluristratified epithelia morphogenesis/maintenance in mice was revealed by the severe phenotype of p63-deficient mice, born with a defect in all pluristratified epithelia [2], [3]. Nevertheless, a key question remained regarding the precise role of p63 in such epithelial developments. Indeed, the presence of uncommitted, single layered, primitive ectodermal cells covering the body surface of p63-deficient mice suggested that the p63-null phenotype leads to an absence of progression along the epidermal lineage with an early block in the epithelial stratification program [2], [10]. In that case, the expression of p63 must occur very early during epidermal development before the stratification program will commit ectodermal cells into epidermal cells, as already suggested by Koster and Roop [10]. Accordingly, in our cell model, the expression of the ΔNp63 isoform occurred very early along the development pathway, soon after the ectodermal cell fate was induced by BMP-4 treatment and prior to the formation of K14^+^/K5^+^ cells. Interestingly, modulation of ΔNp63 expression in zebrafish embryos has demonstrated its role in early dorsal/ventral ectodermal patterning as well as neural/epidermal specification: *in vivo* inhibition of p63 was shown to prevent skin formation, while its exogenous expression promoted epidermal specification [21], [22]. Furthermore, it has been recently reported that in an organotypic reconstitution assay, p63-knockdown keratinocytes dedifferentiated toward an ectodermal fate, as they expressed *de novo* K8/K18 genes, and became unable to stratify [23]. Moreover, ectopic expression of p63 was shown to be able to initiate a stratification program in single-lung epithelia, as demonstrated by histological and molecular analyses [10]. All this strengthens the notion that K14^+^/K5^+^ epithelial cells can only be produced when ΔNp63 is expressed, as clearly demonstrated in the present study.

On the other hand, clumps of terminal-differentiated cells in epidermis of p63-deficient mice were observed by McKeon et al. [3], [6], [9]. Thus, the authors suggested that the phenotype of these mice was not due to a default in embryonic epidermal differentiation but resulted from the inability of the epithelial stem cells to self renew. Accordingly, they recently demonstrated that ΔNp63 is required for adult epithelial stem cells to maintain their proliferative potential [6]. We believe that, instead of a contradiction, our data along with those described by Roop's team, strongly support a dual function for ΔNp63 during embryonic epidermal fate and in epithelial homeostasis. Indeed, self renewal deficiency in absence of p63 cannot explain that embryonic commitment of thymic epithelial cells is not affected in p63-deficient mice [6], [8]. Furthermore, these thymic p63-deficient epithelial cells do not express K14^+^/K5^+^ but K5^+^/K18^+^ and cannot therefore be considered as equivalent of epidermal stem cells. It suggests that mechanisms, in addition to the proliferative capability, are affected by the absence of p63 in order to explain the lack of K5^+^/K14^+^ epidermal cells in our *in vitro* model, as well as *in vivo* in p63-null mice.

Finally, we observed in absence of p63, the presence of structures composed of epithelial cells positive for K5 and K18. Interestingly, as for thymic K5^+^/K8^+^ epithelial cell commitment shown in p63-deficient mice [6], the differentiation of these ES-derived K18/K5-positive cells was not inhibited by the absence of p63. Altogether, these and our results suggest that epithelial lineages are differently affected by the absence of p63 during their morphogenesis. Distinct functions that might be activated sequentially during embryogenesis and the adult life span can then be attributed to p63. It is clear that p63 will have a major contribution in epithelial cell development through its regulatory impact on stem cell proliferative potential [6]. But it also appears that p63 may contribute to the required molecular steps implicated in other epithelial cell commitments, a role which is in accordance with the severe phenotype described for the p63-deficient mice. In this regard, we propose that, during embryogenesis, the ectodermal inducing phase (triggered by BMP-4 in our system) must include, as a necessary step, the expression of ΔNp63 to allow pluristratified epithelial cell fate to occur.

## Materials and Methods

### Cell culture and neuro-ectodermal differentiation

The mouse ES cell line CGR8 and derived clones were maintained as previously described [13]. Briefly, the ES cells were routinely cultured in flasks coated with PBS 0.1% gelatin, in GMEM medium (Gibco BRL, Invitrogen, Cercy-Pontoise, France) supplemented with 10% FCS (FCIII, Perbio-Hyclone, Bezons, France), 1 mM non-essential amino acids, 1 mM Sodium Pyruvate, 0.1 mM ß-mercaptoethanol and 10^3^ U/ml LIF (Leukemia Inhibitory Factor, our own production), and passaged every 2 days. The ES-derived ectodermal cell line was routinely cultured in DMEM medium (Gibco BRL) supplemented with 10% FCS (FCII, Hyclone) and 0.1 mM βME, and passaged every 2 days. For the differentiation protocol, NIH-3T3 cells were grown to confluency in DMEM 10% FCS, they were fixed with PBS 3% formaldehyde (Sigma-Aldrich, France) for 15 min, washed with PBS and incubated with glycine (1 mM) (ICN Pharmaceuticals, France) for 15 min to saturate free formaldehyde sites. Dishes or coverslips were stored in PBS at 4°C until used for ES cell differentiation. For neuro-ectodermal differentiation, ES cells were cultured on fixed NIH-3T3 cells for 9 days in ES cell culture medium in which serum was replaced with 10% Knock-out Serum Replacement (KSR) (Gibco BRL). At day 9 and till day 14, the differentiation medium (10% KSR) was replaced by the ES cell culture medium (10% FCS, without LIF addition). When indicated, 0.5 nM human recombinant BMP-4 (R&D Systems Europe, UK) was added to the differentiation medium each day from day 3 to day 5.

### Antibodies and immunofluorescence

Cells were fixed in methanol at −20°C for 15 min, incubated with PBS containing 2.5% bovine serum albumin (BSA) (Sigma-Aldrich) and 2.5% normal donkey serum (Jackson Immunoresearch) for 30 min and stained as already described [13]. The primary antibodies used for immunofluorescence were: rabbit anti-ΔNp63 (Poly6190, Biolegend, Ozyme, France), rabbit anti-mouse K5 (PRB-160P, Babco, Covance, USA), rabbit anti-mouse K14 (PRB-155P, Babco), rabbit anti-mouse K10 (PRB-159P, Covance), rabbit anti-involucrin (PRB-140C-0200, Covance, Eurogentec, Belgium), rabbit anti-luciferase (AB-21176, Abcam) and rabbit anti-Pax-6 (Abcyss, Paris, France); monoclonal anti-mouse K14 (NE12 8EW, Novocastra Laboratories Ltd), anti-mouse K18 (MAB 3234, Millipore, France), anti-Flag C-myc (9E10, Santa Cruz Biotechnology, Tebu International, France). Secondary Alexa-488 and Alexa-594 donkey anti-mouse and anti-rabbit antibodies were purchased from Molecular Probes (Invitrogen).

### Real-time quantitative RT-PCR

RNAs were extracted using Trizol reagent (Invitrogen) and incubated with DNase I (Ambion, France), according to the manufacturer's instructions to minimize genomic DNA contamination. cDNAs were synthesized using the Superscript II Reverse Transcriptase (Invitrogen). The relative expression level of transcripts was quantified by q-real time-PCR using the Power SYBR-Green PCR Master Mix (Perkin Elmer Applied Biosystems, France) on an ABI PRISM 7500 Sequence Detection System, according to the manufacturer's instructions. The expression of each gene was determined relative to 36B4 as an internal control and the fold stimulation was calculated by using the equation (2)^−ΔΔCT^, where ΔΔCT = CT gene – CT 36B4 and ΔΔCT = ΔCT stimulated condition – ΔCT unstimulated condition. Each gene was amplified using the appropriate specific primers.

**Table:** 

36B4:	5′-TCCAGGCTTTGGGCATCA-3′	5′-CTTTATCAGCTGCACATCACTCAGA-3′
BMP-2:	5′-TCAAGCCAAACACAAACAGC-3′	5′-ACCCCACATCACTGAAGTCC-3′
BMP-7:	5′-GGAAAAATGTCTGCCAGGAA-3′	5′-AGGCTTGCGATTACTCCTCA-3′
ΔNp63:	5′-TGTACCTGGAAAACAATGCCCA-3′	5′-GACGAGGAGCCGTTCTGAATCT-3′
FGRF2b:	5′-CACCAACTGCACCAATGAAC-3′	5′-TGCTTGAATGTGGGTCTCTG-3′
GATA3:	5′-CCGAAACCGGAAGATGTCTA-3′	5′-GTTGAAGGAGCTGCTCTTGG-3′
IKKa:	5′-GGAAAGTATGGGCTGAAGCA-3′	5′-CTGCTCTTTGTCCCTGGAAG-3′
Involucrin:	5′-CACAATGCCAGGTCTTCACTGA-3′	5′-AGGGTTTGGCCGCTTCTC-3′
Jagged 1:	5′-TCTCTGACCCCTGCCATAAC-3′	5′-CAGCCTGGAGAACACTCACA-3′
K1:	5′-AACCCGGACCCAAAACTTAG-3′	5′-CCGTGACTGGTCACTCTTCA-3′
K10:	5′-GACAACTGACAATGCCAACG-3′	5′-CAGGGTCACCTCATTCTCGT-3′
K14:	5′-AAGGTCATGGATGTGCACGAT-3′	5′-CAGCATGTAGCAGCTTTAGTTCTTG-3′
K5:	5′-CCTGCAGAAGGCCAAGCA-3′	5′-TGGTGTTCATGAGCTCCTGGTA-3′
K18:	5′-AATCGAGGCACTCAAGGAAGAA-3′	5′-GGCATCCACTTCCACAGTCA-3′
Filaggrin:	5′-CTGTTCCTGCAATAGGACTGAC-3′	5′-TGCTGAAGAAAGGGCAGA-3′
Lam A3:	5′-CTGCCACCCTCAGTTCACAGT-3′	5′-CGCCGTACGATGATACCTTATCA-3′
Lam B3:	5′-AAGACCCTTCAGGAGCCTTC-3′	5′-TGGCTCAGCAGGCTAGAACT-3′
Lam C2:	5′-GGCCCTTCGAGACATTCTAA-3′	5′-GTCGTTCTCTTGGCTCCTTG-3′
Notch1:	5′-GCAATCTCAAGTCTGCCACA-3′	5′-GCTTCCTTGCTACCACAAGC-3′
Perp:	5′-GGATGGGAGGATGGACTAGG-3′	5′-GTGACAGTGGTGACCCTCCT-3′
TAp63:	5′-TGGATGAACCTTCCGAAAATG-3′	5′-TGCGGATACAATCAATGCTAAT-3′

### Lentiviral vector production

The lentiviral vector production was performed using 293-FT producing cells, cultured in 10% FCS DMEM medium on collagen matrix, and transfected in Lipofectamine 2000 with the plasmids required for the lentivirus production (MD2G, 8.93, 8.92 vectors) and expression plasmids for c-myc-tagged-ΔNp63, Pax6 or red-tomato fluorescent protein. On the day following transfection, DMEM medium was replaced with Opti-MEM supplemented with 25 mM Hepes, 0.1 mM Non Essential Amino acids and 1 mM Sodium Pyruvate. Forty-eight later, the supernatants were collected and concentrated using Centricon Plus-20 columns (Millipore).

### Plasmid DNA Transfection: Generation of stable ES Shp63 clones

Plasmid pSuper vectors containing short-hairpin (sh) p63 or control sequence were kindly provided by C. Missero (CEINGE Biotecnologie Avanzate Scarl, Napoli). A plasmid c-myc-tagged-ΔNp63 vector was kindly provided by H. Van Bohkoven (Nijmegen, Netherlands). Undifferentiated ES cells (passage 25) were transfected with Lipofectamine 2000 (Invitrogen) according to the manufacturer instructions. Twenty-four hours later, transfected ES cells were passaged and treated 12 hours later with puromycin for 15 days. Resistant ES stable clones were isolated and cultured separately in the presence of puromycin (1 µg/ml). Isolated clones were tested by immunoblotting with anti-ΔNp63, either for expression of ΔNp63 (ES cell stably expressing ΔNp63) or for expression of the sh-p63 after transfection with the c-myc-tagged-ΔNp63 expression vector.

### FACS analysis

FACS analysis was performed as previously described [13] with anti-mouse K14 (NE12 8EW) or K5 (PRB-160P) antibodies. Briefly, ES cells cultured on fixed NIH-3T3 cells were detached with trypsin 0.5 mM EDTA. They were fixed in 2% formaldehyde in PBS, permeabilized in PBS-0.5% saponin (Sigma-Aldrich)-0.5% BSA for 10 min and saturated with PBS-0.5% saponin-0.5% BSA-0.5% rabbit or swine serum depending on secondary antibodies for 30 min. The ES cells were stained with primary mAbs for 45 min. Mouse isotype control mAb (PharMingen) and control rabbit serum was used to set the background level of fluorescence. Rabbit anti-mouse and swine anti-rabbit coupled FITC antibodies (Dakko A/S, country) were added for 45 min. Cells were monitored by flow cytofluorimetry on a FACScan system using CellQuest software (BD Biosciences).

### Immunoblotting analysis

HeLa cells were cultured in 60 mm dishes and transfected with TAp63 (α, β, γ) and ΔNp63 (α, β, γ) expressing vectors, and lysed 24 hours later in buffer containing 25 mMTris HCl pH 7.6, 150 mM NaCl, 1% NP-40, 1% sodium deoxycholate, 1% SDS. Differentiating ES cells were lysed at different time points in the same buffer. Undifferentiated ES cells were cultured in 60 mm dishes, transfected with ΔNp63-c-myc-tag expressing vector, and lysed 48 h later in buffer containing 50 mM Tris pH 7.4, 150 mM NaCl, 1% Triton X-100, 10 µM leupeptin, 1 mM AEBSF, 100 units/ml aprotinin, 10 mM NaF and 1 mM Na_3_VO_4_. Samples (30 µg of proteins) were resolved by 12% SDS-PAGE, transferred to Hybond C Extra nitrocellulose membranes (Amersham Biosciences, France), and then incubated with the appropriate antibodies: mouse monoclonal antibody raised against p63 (4A4, Sc-8431, Santa Cruz Biotechnology, Tebu International), mouse monoclonal IgG2b antibody raised against Erk-2 (Sc-1647, Santa Cruz Biotechnology, Tebu International) and rabbit polyclonal antibodies raised against ΔNp63 (Poly6190). Proteins were visualized with the ECL system (Amersham) using HRP-conjugated anti-rabbit or anti-mouse secondary antibodies.

## Supporting Information

Figure S1 Schematic graph representing the ES cell differentiation protocol used in this study. Mouse ES cells were seeded in culture on NIH-3T3-PFA-fixed cells in serum-free medium for 14 days, with change of medium every 2 to 3 days. (A) In the absence of BMP-4 treatment, ES cells differentiate exclusively into neural cell populations. (B) Treatment with BMP-4 from day 3 to day 5 inhibits neural cell fate and induces K18-positive ectodermal cell differentiation. When serum is added at day 9, ectodermal cells differentiate further into epithelial cells expressing K5 and K14 basal squamous stratified epithelium markers, as well as K1 and K10 terminal differentiation markers.
(1.86 MB TIF)

Figure S2 DNp63 expression during ES cell differentiation. (A) Time course expression of K18 and K14 genes. Relative K18 and K14 gene expression levels were determined by real-time RT-PCR at the indicated time points during the ES cell differentiation. The value for each gene was normalized to the untreated control cells at day 1. (B) Percentages of K18-positive cells obtained at day 7 and 14 from ES cell cultures treated or not with BMP-4 as determined by flowcytometric analysis. (C) Time course expression of DNp63 and 36B4 genes. The graph represents the Ct values for real-time RT-PCR analysis of the DNp63 and the 36B4 (control) mRNA expression at the indicated time points during the ES cell differentiation and from 3T3 fibroblasts and NMK. This graph displays one representative experiment out of three. (D) Proteins extracted from HeLa cells transfected with p63 isoforms, NMK and myocytes were immunoblotted with anti-p63 and anti-Erk2 (as loading control) antibodies. NMK (known to express endogenous DNp63 isoforms) and differentiating myocytes (which have been shown to express the TAp63 isoform, [24]) served as molecular weight references.
(2.05 MB TIF)

Figure S3 The ectodermal differentiation is not affected by the absence of p63.(A) Immunofluorescence staining (left panel) for K18 at day 14 of differentiation (BMP-4 and serum treatments) in control- and p63-sh ES cell clones. Bars = 50 µm. (B) Real-time RT-PCR analysis, at day 7 (BMP-4 treatment) and day 14 (BMP-4 and serum) of differentiation for K18 gene expression from ES cell clones expressing either a control- or p63- shRNA. The values were normalized to untreated control cultures and represent the average of three experiments±sd.
(3.62 MB TIF)
