# Supplementary figures and images for: ΔNp63 Is Essential for Epidermal Commitment of Embryonic Stem Cells
Source: PLoS One. 2008 Oct 17;3(10):e3441. doi: 10.1371/journal.pone.0003441 (PMC2562986; doi:10.1371/journal.pone.0003441)

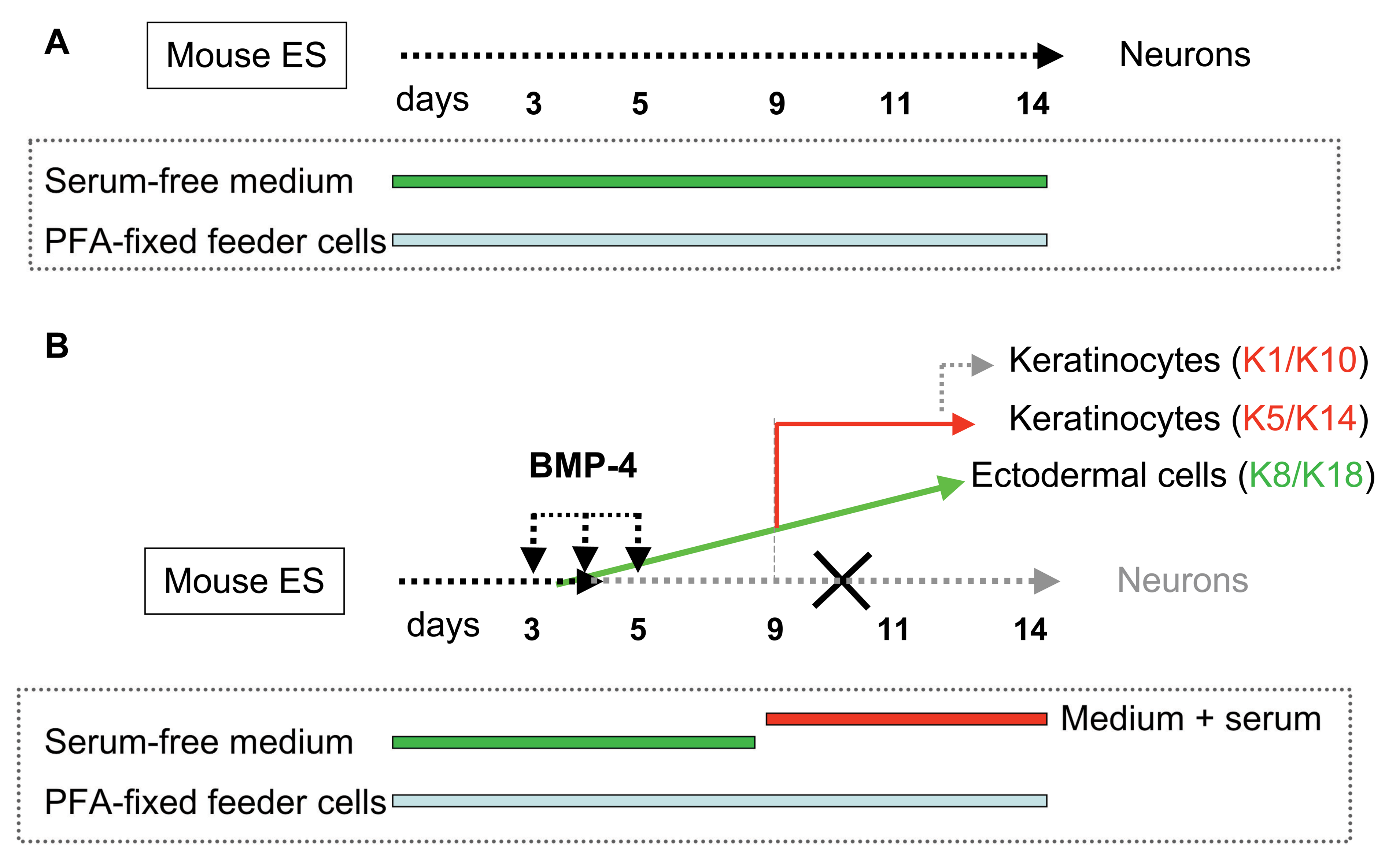

Supplement: Figure S1 — Schematic graph representing the ES cell differentiation protocol used in this study. Mouse ES cells were seeded in culture on NIH-3T3-PFA-fixed cells in serum-free medium for 14 days, with change of medium every 2 to 3 days. (A) In the absence of BMP-4 treatment, ES cells differentiate exclusively into neural cell populations. (B) Treatment with BMP-4 from day 3 to day 5 inhibits neural cell fate and induces K18-positive ectodermal cell differentiation. When serum is added at day 9, ectodermal cells differentiate further into epithelial cells expressing K5 and K14 basal squamous stratified epithelium markers, as well as K1 and K10 terminal differentiation markers. (1.86 MB TIF) [file pone.0003441.s001.tif]

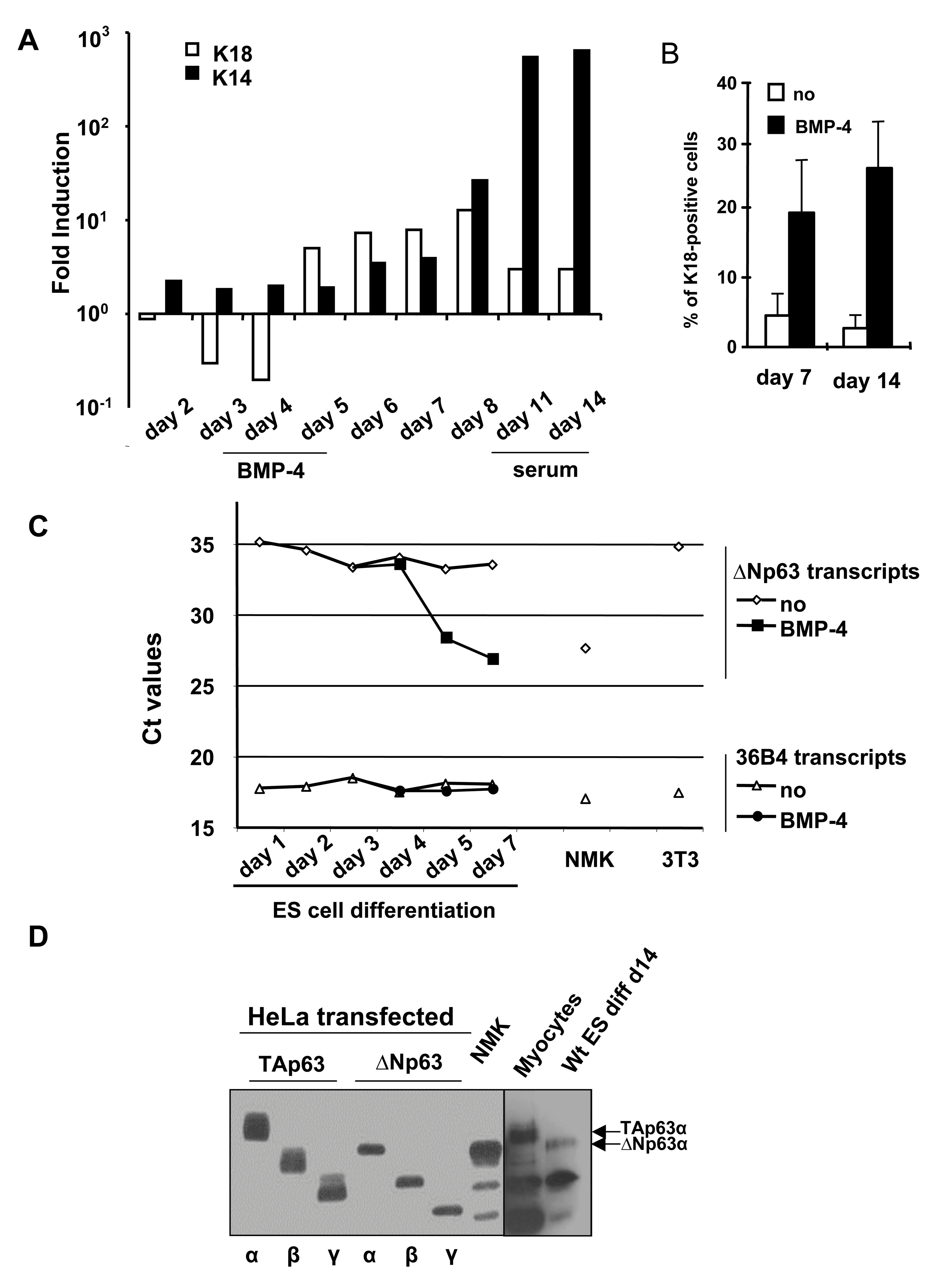

Supplement: Figure S2 — DNp63 expression during ES cell differentiation. (A) Time course expression of K18 and K14 genes. Relative K18 and K14 gene expression levels were determined by real-time RT-PCR at the indicated time points during the ES cell differentiation. The value for each gene was normalized to the untreated control cells at day 1. (B) Percentages of K18-positive cells obtained at day 7 and 14 from ES cell cultures treated or not with BMP-4 as determined by flowcytometric analysis. (C) Time course expression of DNp63 and 36B4 genes. The graph represents the Ct values for real-time RT-PCR analysis of the DNp63 and the 36B4 (control) mRNA expression at the indicated time points during the ES cell differentiation and from 3T3 fibroblasts and NMK. This graph displays one representative experiment out of three. (D) Proteins extracted from HeLa cells transfected with p63 isoforms, NMK and myocytes were immunoblotted with anti-p63 and anti-Erk2 (as loading control) antibodies. NMK (known to express endogenous DNp63 isoforms) and differentiating myocytes (which have been shown to express the TAp63 isoform, [24]) served as molecular weight references. (2.05 MB TIF) [file pone.0003441.s002.tif]

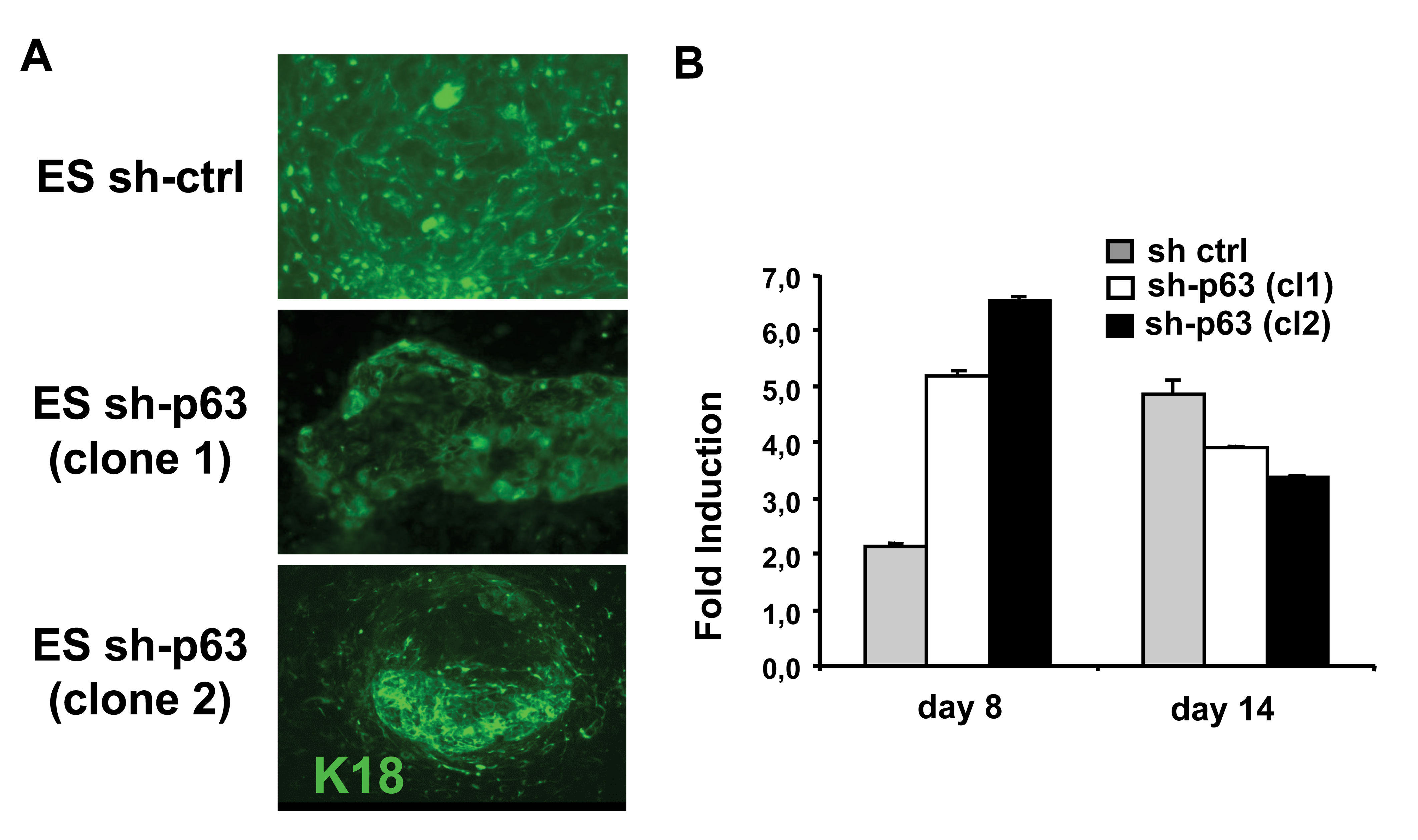

Supplement: Figure S3 — The ectodermal differentiation is not affected by the absence of p63.(A) Immunofluorescence staining (left panel) for K18 at day 14 of differentiation (BMP-4 and serum treatments) in control- and p63-sh ES cell clones. Bars = 50 µm. (B) Real-time RT-PCR analysis, at day 7 (BMP-4 treatment) and day 14 (BMP-4 and serum) of differentiation for K18 gene expression from ES cell clones expressing either a control- or p63- shRNA. The values were normalized to untreated control cultures and represent the average of three experiments±sd. (3.62 MB TIF) [file pone.0003441.s003.tif]
